# Preparation of heterostructured WO_3_/TiO_2_ catalysts from wood fibers and its versatile photodegradation abilities

**DOI:** 10.1038/s41598-017-01244-y

**Published:** 2017-04-24

**Authors:** Likun Gao, Wentao Gan, Zhe Qiu, Xianxu Zhan, Tiangang Qiang, Jian Li

**Affiliations:** 1 Key Laboratory of Biobased Material Science & Technology (Education Ministry), Northeast Forestry University, Harbin, 150040 P.R. China; 2 Research Center of Wood Bionic Intelligent Science, Northeast Forestry University, Harbin, 150040 P.R. China; 3 Dehua TB New Decoration Material Co., Ltd, Huzhou, 313200 P.R. China

## Abstract

A facile route was adopted to synthesize heterostructured WO_3_/TiO_2_ photocatalysts from wood fibers through a two-steps hydrothermal method and a calcination process. The prepared WO_3_/TiO_2_-wood fibers were used as photocatalysts under UV irradiation for photodegradation of rhodamine B, methylene blue and methyl orange. In calcination process, the wood fibers acted as carbon substrates to prepare the WO_3_/TiO_2_ photocatalysts with high surface area and unique morphology. Thus, the significant enhanced photodegradation efficiency of the organic pollutants with the WO_3_/TiO_2_-wood fibers under UV irradiation was obtained. The photodegradation rates are measured which confirms the highest performance of the WO_3_/TiO_2_-wood fibers after calcination in comparison to the TiO_2_-wood fibers after calcination and the pure WO_3_/TiO_2_ after calcination. Moreover, the photodegradation efficiency of the WO_3_/TiO_2_-wood fibers after calcination under visible light is high. Our results demonstrated that the WO_3_/TiO_2_-wood fibers after calcination are a promising candidate for wastewater treatment in practical application.

## Introduction

There has been an increasing environmental problem in recent years due to global warming. From the viewpoint of the utilization of solar energy, many efforts have been devoted to develop the light-driven photocatalysts, which could decompose harmful chemicals existed in the environment by using sunlight^1^. Photocatalysis is used to remove pollutants through photo-oxidation in the presence of light and photocatalyst, which can convert optical energy into the energy for chemical reactions^2^. Semiconductor materials such as ZnO, TiO_2_, SnO_2_, and WO_3_, have been extensively studied as catalysts to degrade environment pollutants owing to their strong oxidative abilities, suitable band gaps and excellent stabilities in water solution^3–8^. In practical, photocatalysts that are more stable, more efficient, non-toxic and capable of harvesting sunlight are highly desirable.

Markedly, TiO_2_ semiconductor with the band gap of 3.2 eV is a promising photocatalyst and widely used in photodegradation of aqueous or gaseous toxic organic pollutants for water treatment and air purification due to its nontoxicity and environmental friendly properties along with stability^9^. In the photodegradation process of toxic contaminants using TiO_2_, the whole process could be insured facile and environment-friendly, which the reaction could happen at ambient temperature and pressure, and the reaction products are usually CO_2_ and H_2_O. It is worth noting that how to enhance photocatalytic efficiency of photocatalysts is a basic and important task, not merely for the theoretical significance but also for the advance in applications.

For the purpose of reducing the degradation and enhancement in catalytic properties, many research reported on the enhancement of toxic contaminants degradation through doping TiO_2_ with extrinsic dopants such as metal oxide or metal elementary^10, 11^. For example, after doping with V, Pt, Ag, and Au, TiO_2_ semiconductor films showed obvious increase in catalytic properties^12–15^. Liang *et al*. reported that Ce/TiO_2_ and Ag/TiO_2_ showed higher photocatalytic degradation efficiency of formaldehyde gas under UV irradiation^16^. Tungsten oxide (WO_3_), as an important n-type semiconductor with a narrow gap (∼2.8 eV), has drawn much attention for its unique electronic, chemical and optical properties^17, 18^. Herein, WO_3_ was selected as potential dopant to decorate the pure TiO_2_. For the photodegradation, coupling TiO_2_ with WO_3_ can extend the optical absorption to the visible region to enhance the photocatalytic efficiency^19–21^. The edge of the valence band and conduction band in WO_3_ are lower than that in TiO_2_. The differences in band edge position in the WO_3_/TiO_2_ photocatalyst created potential gradient at the composite interface, which would facilitate the charge separation and inhibited charge carrier recombination^22^.

Generally, people considered that wooden materials are always used to light a fire, and it is attributed to carbon and other components originated from cellulose, hemicellulose and lignin. In the synthesis process of photocatalysts, the presence of carbon in the wood as the substrate can lead to a relatively high surface area photocatalysts with respect to pure photocatalysts^23^. Generally, the specific architectures can be obtained using hard, sacrificial or soft templates such as wood, textiles and so on^24, 25^. Moreover, the abundant pores originated from wood intrinsic properties are beneficial to absorb gas, on the other hand, it containing plentiful hydroxide radical has emerged as a better substrate material for the growth of metal-oxide semiconductor films such as TiO_2_^26^, WO_3_^27^, CoFe_2_O_4_^28, 29^ and Cu_2_O^30^.

Herein, the enhanced photodegradation of organic pollutant on heterostructured WO_3_/TiO_2_ photocatalysts from wood fibers through a two-steps hydrothermal method and a calcination process is assumed and reported. The photodegradation of rhodamine B, methylene blue and methyl orange on the samples under UV irradiation are discussed in details.

## Results

**Figure 1:**
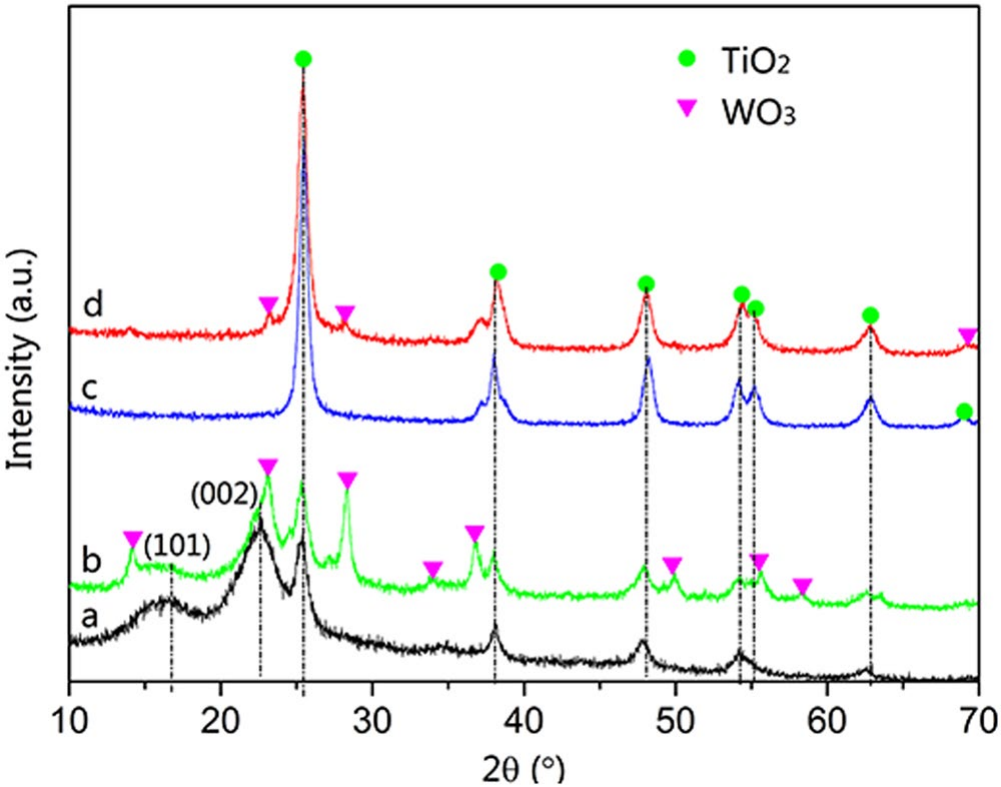
XRD patterns of (**a**) the TiO_2_-wood fibers, (**b**) the WO_3_/TiO_2_-wood fibers, (**c**) the TiO_2_-wood fibers after calcination and (**d**) the WO_3_/TiO_2_-wood fibers after calcination.

Figure 1 presents the XRD patterns of the TiO_2_-wood fibers, the WO_3_/TiO_2_-wood fibers, the TiO_2_-wood fibers after calcination and the WO_3_/TiO_2_-wood fibers after calcination. In Fig. 1a,b, the diffraction peaks at 14.8° and 22.5° belong to the (101) and (002) crystal planes of cellulose in the wood^31^. It can be found that all the diffraction peaks in Fig. 1 are well indexed to the standard diffraction pattern of anatase TiO_2_ (JCPDS file No. 21-1272)^32^ and WO_3_ (JCPDS file No. 75-2187)^33^, indicating that the present synthesis strategy successfully achieves WO_3_/TiO_2_ heterostructures with high crystallinity on wood substrate. In Fig. 1a,c, the diffraction peaks at 25.5°, 38.0°, 48.3°, 54.2°, 55.3°, 62.9° and 69.0° can be perfectly identified to (101), (004), (200), (105), (211), (204) and (116) crystal planes of anatase TiO_2_, respectively^32^. The curves in Fig. 1b,d show that all of the new diffraction peaks of the WO_3_/TiO_2_-wood fibers and the WO_3_/TiO_2_-wood fibers after calcination center at 2*θ* = 14.2°, 23.1°, 28.4°, 33.8°, 36.9°, 49.9°, 55.7° and 58.4° except the diffraction peaks of TiO_2_, are agree with (100), (001), (200), (111), (201), (220), (221) and (400) planes of pure hexagonal WO_3_^33, 34^. Moreover, in Fig. 1c,d, the sharper diffraction peaks of WO_3_ and TiO_2_ suggest that calcination at 500 °C for 3 h is sufficient to crystallize pure anatase TiO_2_ and hexagonal WO_3_ nanostructures.

**Figure 2:**
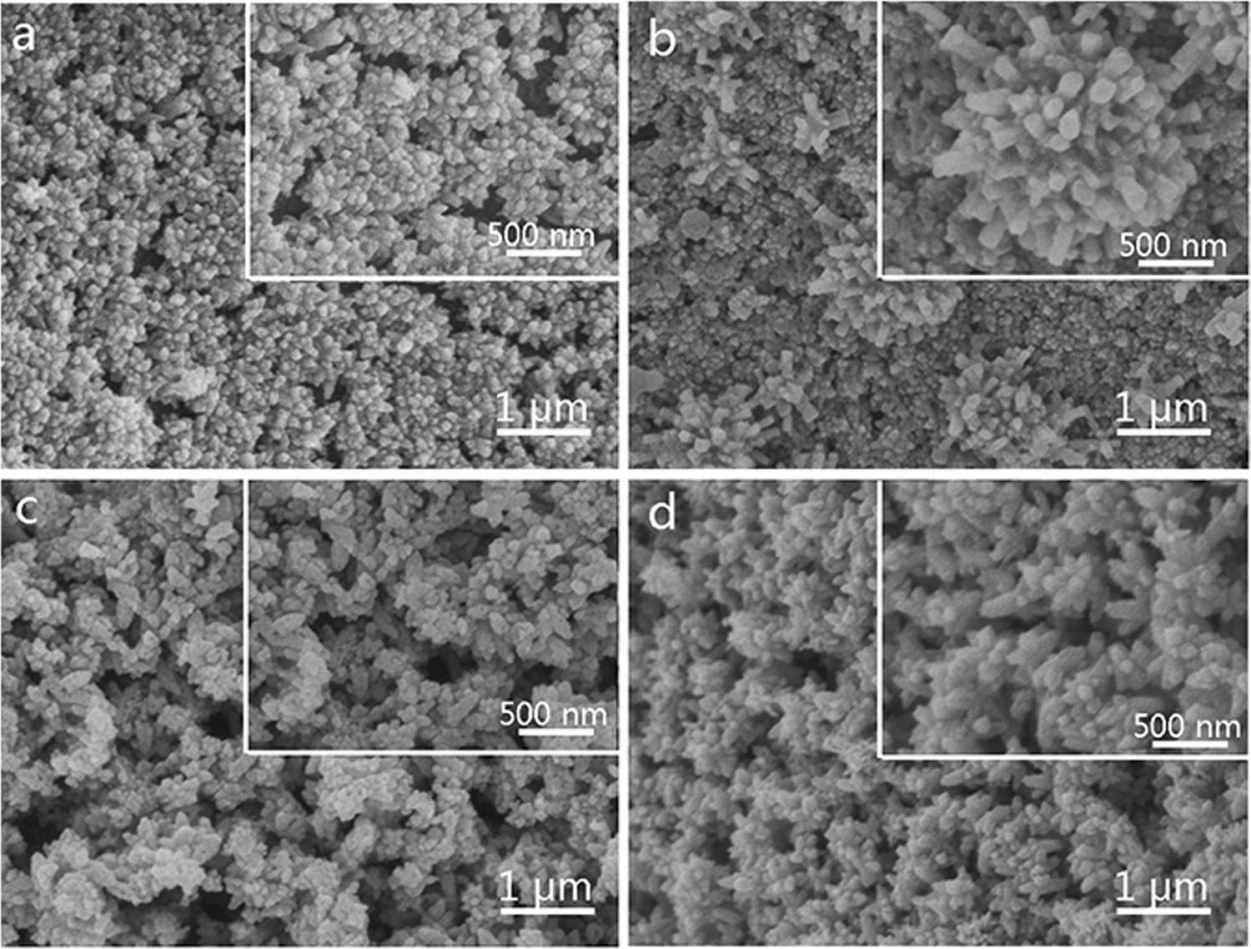
SEM images of (**a**) the TiO_2_-wood fibers, (**b**) the WO_3_/TiO_2_-wood fibers, (**c**) the TiO_2_-wood fibers after calcination and (**d**) the WO_3_/TiO_2_-wood fibers after calcination. (The inserts show the SEM images of each sample at high magnifications).

In Fig. 2, the SEM images were utilized to investigate the morphologies of the samples. It is obvious that spherical particles of TiO_2_ in the TiO_2_-wood fibers (Fig. 2a) are converted into rhombic type structures after calcination (Fig. 2c). As shown in Fig. 2b,d, the number of actinomorphic WO_3_ flowers loaded on the surfaces of the TiO_2_ spherical particles increases, which is due to the calcination at 500 °C for 3 h. Compared to the morphologies of the samples before calcination in Fig. 2a,b, the samples structures after calcination in Fig. 2c,d become more compact. This suggests formation of nanoparticles with high aspect ratio and further growth of these nanostructures in the case of the presence of wood fibers after calcination.

**Figure 3:**
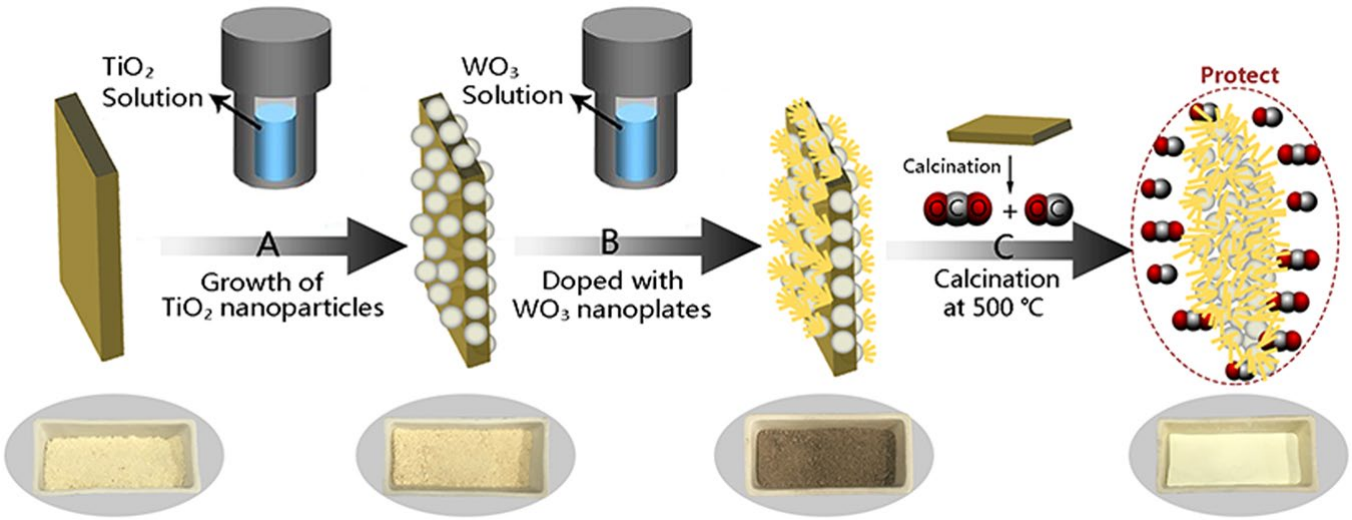
Synthesis scheme of the WO_3_/TiO_2_ catalysts from wood fibers.

Based on the above results, the synthesis process of the WO_3_/TiO_2_ catalysts from wood fibers is illustrated in Fig. 3. After two-steps hydrothermal synthesis (A and B), the wood fibers were coated by WO_3_/TiO_2_ films consisted of actinomorphic WO_3_ flowers and TiO_2_ spherical particles. According to statistics, the carbon contents in the cellulose of wood are about 40∼50%, while the cellulose is the main components of wood and the cellulose contents are about 50%. Thus, during calcination in air (C), the carbon in the wood fibers would be oxidized to CO and CO_2_ gases. CO and CO_2_ gases can act as reductive gases to protect the materials structures in calcination. After calcination, the wood fibers were removed leading the WO_3_ flowers and TiO_2_ spherical particles become more compact.

**Figure 4:**
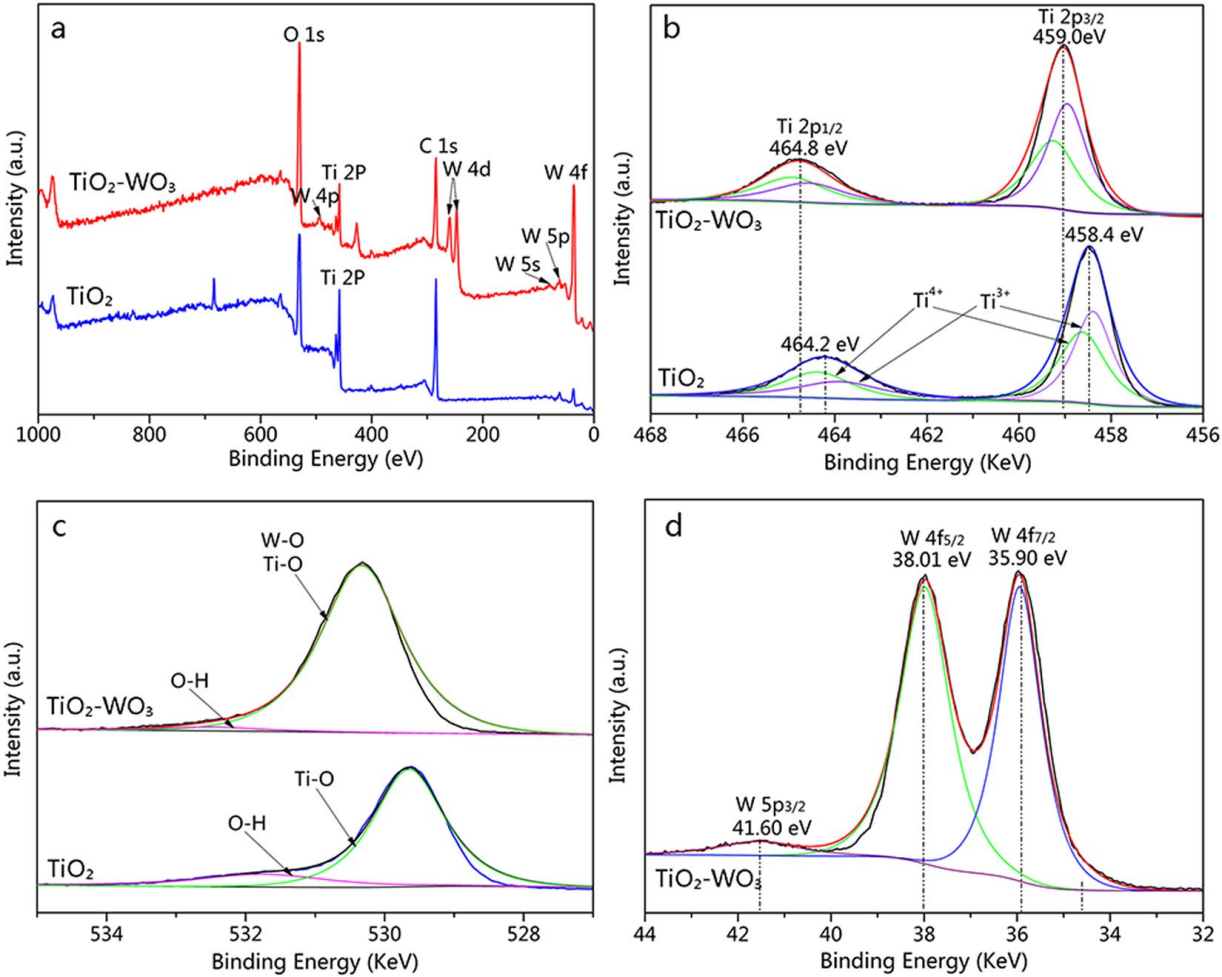
(**a**) Survey scan and (**b**) peaking-fitting results of Ti 2p XPS spectra of the TiO_2_-wood fibers after calcination and the WO_3_/TiO_2_-wood fibers after calcination, (**c**) peaking-fitting results of O 1 s XPS spectra of the TiO_2_-wood fibers after calcination and the WO_3_/TiO_2_-wood fibers after calcination and (**d**) W 4 f XPS spectra of the WO_3_/TiO_2_-wood fibers after calcination.

For the sake of clarification of the surface chemical composition and electronic structure, XPS analysis was applied to characterize the TiO_2_-wood fibers after calcination and the WO_3_/TiO_2_-wood fibers after calcination. The wide-scan spectra in Fig. 4a show that the Ti, O, and C elements exist on the TiO_2_-wood fibers after calcination and the Ti, O, W, and C elements exist on the WO_3_/TiO_2_-wood fibers after calcination. Moreover, trace amounts of carbon, which originates from the residual carbon in the structure and the adventitious hydrocarbon in the XPS instrument itself.

The Ti 2p spectrum of the TiO_2_-wood fibers after calcination in Fig. 4b shows two peaks at binding energies of 458.4 eV and 464.2 eV, which correspond to the Ti 2p_3/2_ and Ti 2p_1/2_ peaks, respectively. The gap between Ti 2p_3/2_ and Ti 2p_1/2_ lines is 5.8 eV, suggesting the existence of the Ti^4+^ oxidation state^9^. And the binding energy of Ti 2p_3/2_ in the TiO_2_-wood fibers after calcination can be fitted into two components, one located at 458.67 eV, assigned to a Ti^4+^ species, and the other located at 458.37 eV, attributed to a Ti^3+^ species. With the incorporation of W species, the intensity of Ti 2p is decreased as indicated in Fig. 4a,b. This also suggests that relatively more W species are present on the surface of the structure. The slight decrease in binding energy in the WO_3_/TiO_2_-wood fibers after calcination suggests that some of Ti^4+^ species are converted to a lower oxidation state, such as Ti^3+^ species; meanwhile it can be observed clearly the binding energy of Ti^3+^ increases. The peaks positions for Ti 2p in the WO_3_/TiO_2_-wood fibers after calcination shift to higher binding energy bands than those in the TiO_2_-wood fibers after calcination. This confirms the chemical condition of TiO_2_ transfers from Ti-O-Ti to Ti-O-W, which means a strong interaction between WO_3_ and TiO_2_ in the WO_3_/TiO_2_-wood fibers after calcination.

In Fig. 4c, the O 1 s band has been greatly modified with the introduction of the W species. For both the two samples, the O 1 s can be fitted with two peaks. The peak at 531.75 eV in the TiO_2_-wood fibers after calcination and the peak at 532.50 eV in the WO_3_/TiO_2_-wood fibers after calcination are related to the hydroxyl groups^19^. For the TiO_2_-wood fibers after calcination, the peak at 529.63 eV is assigned to the oxygen bound to Ti, while the O 1 s region of the WO_3_/TiO_2_-wood fibers after calcination with the peak at 530.33 eV contained contributions from both the Ti-O and W-O is considerably broader. This is because the Ti-O and W-O have similar binding energies^35^.

Figure 4d shows the W 4 f and the W 5p_3*/*2_ core level spectra recorded on the WO_3_/TiO_2_-wood fibers after calcination, and the results of its fitting analysis. To reproduce the experimental data, two doublet functions are used for the W 4 f component and a singlet for the W 5p_3*/*2_ component near 41.60 eV^36^. One doublet contains its highest intensity peak (W 4f_7*/*2_ *)* located near 35.90 eV, which is generated by photoelectrons emitted from tungsten atoms with an oxidation state of +6; i.e. stoichiometric WO_3_. In stoichiometric WO_3_, the W atom has + 6 valence electrons with 5d empty shell (d^0^ oxides). The six valence electrons of the W atom are transferred into the oxygen p-like bands and the oxygen p-like bands are completed filled. Thus, the 5d valence electron is empty and there would be a stronger interaction between the remaining electrons in W atom and the nucleus. That is, the binding energy of W 4 f level of WO_3_ is larger than that of metallic W^37^. And the other peak at 38.01 eV is corresponding to W 4f_5/2_, while the energy gap between the two peaks of W 4f_7/2_ and W 4f_5/2_ is 2.11 belonging to the tungsten in the W^6+^ valance state^36^.

**Figure 5:**
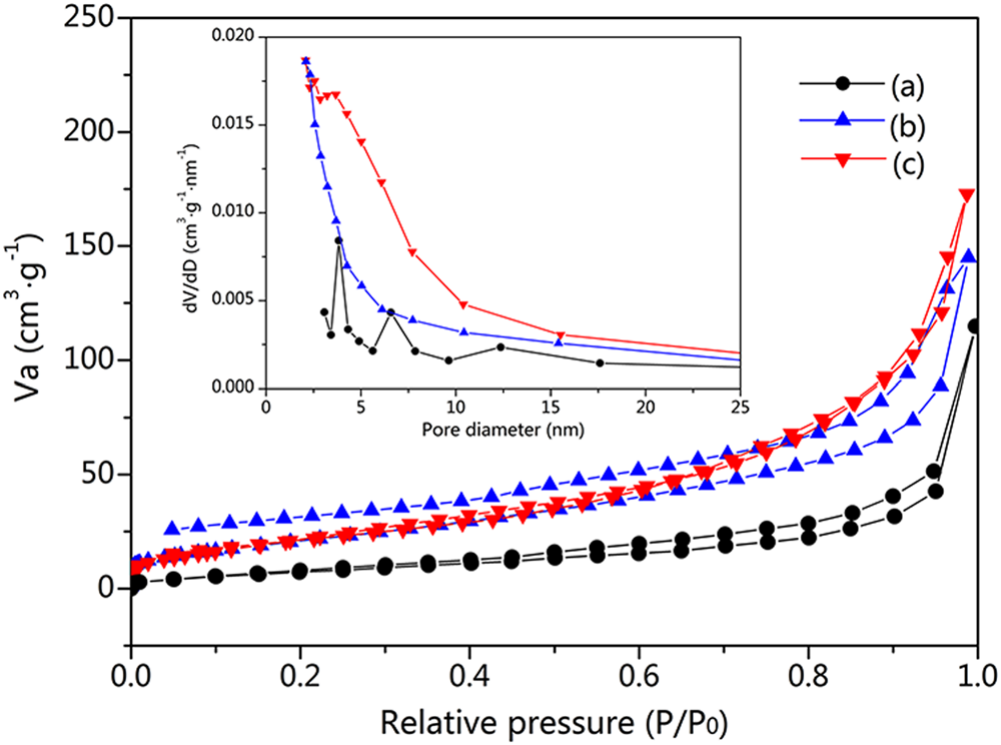
N_2_ adsorption–desorption isotherms of (**a**) the pure WO_3_/TiO_2_ after calcination, (**b**) the TiO_2_-wood fibers after calcination and (**c**) the WO_3_/TiO_2_-wood fibers after calcination. The inset shows the pore size distributions.

To have an insight into the effect of the wood fibers on the porous structure of the samples, BET analysis was carried out. Figure 5 shows the N_2_ adsorption–desorption isotherms of the pure WO_3_/TiO_2_ after calcination, the TiO_2_-wood fibers after calcination and the WO_3_/TiO_2_-wood fibers after calcination. These curves all exhibit small hysteresis loops, which are attributed to type IV isotherms and the representative of mesoporous materials, indicating the presence of mesopores (2–50 nm)^2^. This result is further confirmed by the corresponding pore-size distribution curves (inset in Fig. 5). Furthermore, the isotherm profile of the WO_3_/TiO_2_-wood fibers after calcination shows typical H1 type hysteresis loops in the relative pressure range from 0.4 to 0.9 according to the uniform sized spherical-particles aggregates and hysteresis loops close to H3 type from 0.9 to 1.0, indicating the presence of slit-like pores. The pore size distribution of the WO_3_/TiO_2_-wood fibers after calcination exhibits a broadened pore size range (inset in Fig. 5).

BET surface areas, pore sizes and pore volumes of the WO_3_/TiO_2_-wood fibers after calcination compared to the pure WO_3_/TiO_2_ after calcination and the TiO_2_-wood fibers after calcination are presented in Table 1. From the results shown, it is clear that the preparation of WO_3_/TiO_2_ in the presence of wood fibers after calcination leads to a significantly higher surface area with respect to the pure WO_3_/TiO_2_ obtained in the absence of wood fibers (up to approximately 3.6 times higher). The TiO_2_-wood fibers after calcination without loading WO_3_ lead a relatively low surface area of 81.70 m^2^/g. For samples prepared in the presence of wood fibers *S*_BET_ are higher than 80 m^2^/g. That is, the method used in the study seems to produce a certain heterogeneous system with respect to wood fibers, in terms of the surface properties (surface area, pore size distribution, etc.) of obtained the WO_3_/TiO_2_-wood fibers after calcination. Thus, because of its large surface area, the WO_3_/TiO_2_-wood fibers after calcination provides more photocatalytic reaction sites for the adsorption of reactant molecules and increases the efficiency of the electron–hole separation, so the photocatalytic activity of the WO_3_/TiO_2_-wood fibers after calcination is enhanced.

**Table 1:** The structure parameters of the pure WO_3_/TiO_2_ after calcination, the TiO_2_-wood fibers after calcination and the WO_3_/TiO_2_-wood fibers after calcination.

Sample	BET surface area (m^2^/g)	Pore size (nm)	Pores volume (cm^3^/g)
Pure WO_3_/TiO_2_ after calcination	25.98	3.83	0.17
TiO_2_-wood fibers after calcination	81.70	5.18	0.21
WO_3_/TiO_2_-wood fibers after calcination	92.95	4.98	0.26

**Figure 6:**
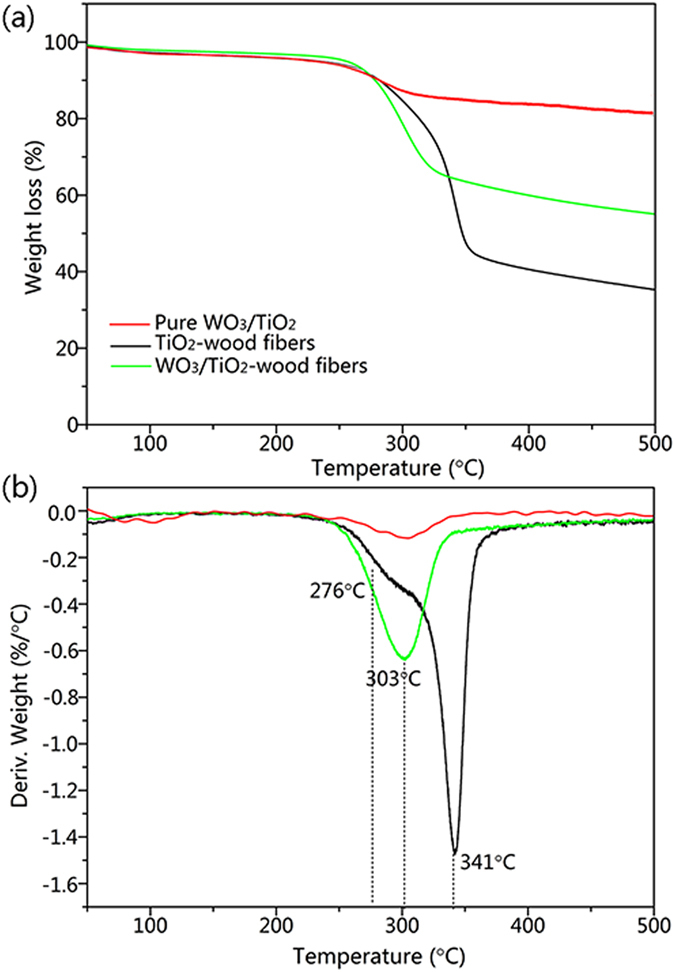
(**a**) TG profiles and (**b**) DTG profiles of the pure WO_3_/TiO_2_, the TiO_2_-wood fibers and the WO_3_/TiO_2_-wood fibers.

The TG and DTG curves of the pure WO_3_/TiO_2_, the TiO_2_-wood fibers and the WO_3_/TiO_2_-wood fibers are shown in Fig. 6. In Fig. 6a, small weight losses appear at about 50–80 °C in the samples, which correspond to a mass loss of physically adsorbed water of approximately 5%. After this peak, the DTG curves of the TiO_2_-wood fibers and the WO_3_/TiO_2_-wood fibers in Fig. 6b shows three decomposition steps: (1) the first decomposition shoulder peak at about 276 °C for the TiO_2_-wood fibers, is attributed to thermal depolymerisation of hemicelluloses or pectin; (2) the major second decomposition peak at about 341 °C and 303 °C for TiO_2_-wood fibers and the WO_3_/TiO_2_-wood fibers, respectively, is attributed to cellulose decomposition. Lignin is the most difficult one to decompose, and its decomposition keeps on along the whole calcination process; (3) the final decomposition process at about 380−600 °C was attributed to all the wood components degradation gradually leading to the aromatization and carbonization. Due to the decomposition of cellulose and lignin, the maximum degradation rates of the WO_3_/TiO_2_-wood fibers become lower than that of TiO_2_-wood fibers. This may be due to the catalysis of WO_3_/TiO_2_ composite film, which generates an accelerated pyrolysis action on wood components.

Moreover, from thermal analysis of samples we obtain information about the evolution of WO_3_/TiO_2_-wood fibers system during calcination. The pure WO_3_/TiO_2_ obtained through hydrothermal synthesis in the absence of wood fibers leads to a total weight loss of about 19.3% after calcination at 500 °C. However, the weight loss of the WO_3_/TiO_2_-wood fibers after calcination at 500 °C is 45.1%, including the losses of carbon and WO_3_/TiO_2_. Thus, it can be calculated that the weight loss of C in the WO_3_/TiO_2_-wood fibers after calcination at 500 °C is 25.8%, that is, the residual C content is about 10.3%.

**Figure 7:**
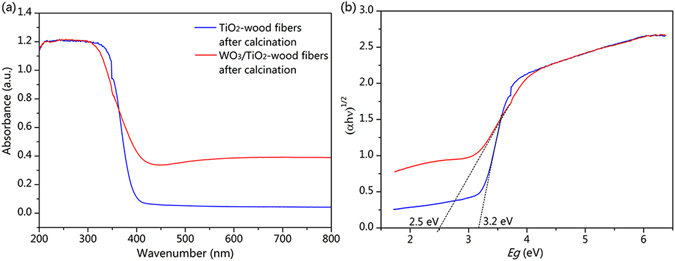
(**a**) UV-vis absorption spectra of the TiO_2_-wood fibers after calcination and the WO_3_/TiO_2_-wood fibers after calcination, and (**b**) the evaluation of the optical band gap using the Tauc plot.

In order to investigate the light absorbance of the samples, the UV−vis diffuse reflection spectra of the TiO_2_-wood fibers after calcination and the WO_3_/TiO_2_-wood fibers after calcination are depicted in Fig. 7a. As for both the TiO_2_-wood fibers after calcination and the WO_3_/TiO_2_-wood fibers after calcination, it presents prominent adsorptions below 380 nm wavelength, whereas the WO_3_/TiO_2_-wood fibers after calcination exhibits a much higher absorption, indicating more intensive effect to UV light. Moreover, the absorption wavelength of the WO_3_/TiO_2_-wood fibers after calcination has a comparatively red shift, indicating that doping with WO_3_ can extend the optical absorption to the visible region. And the TiO_2_-wood fibers after calcination and the WO_3_/TiO_2_-wood fibers after calcination show the absorption edges at about 396 nm and 465 nm, respectively. To calculate valence band position, the optical band gap is determined by the following Tauc equation 1 ^38^:

1 $${(ahv)}^{n}=A(hn-{E}_{g})$$

Where *A* = constant, *hν* = light energy, *E*_*g*_ = optical band gap energy, *α* = measure absorption coefficient, *n* = 0.5 for direct band gap, and *n* = 2 for indirect band gap materials. Because both the TiO_2_ and WO_3_ has direct band gap, the *y* axis of the Tauc plot is (*αhν*)^*1/2*^ for TiO_2_ and WO_3_^39, 40^. In Fig. 7b, the extrapolation of the Tauc plot on *x* intercepts gives the optical band gaps of 3.2 eV and 2.5 eV for TiO_2_ and WO_3_, respectively. Therefore, the conduction band and valence band of WO_3_ are more negative than the corresponding bands of TiO_2_.

**Figure 8:**
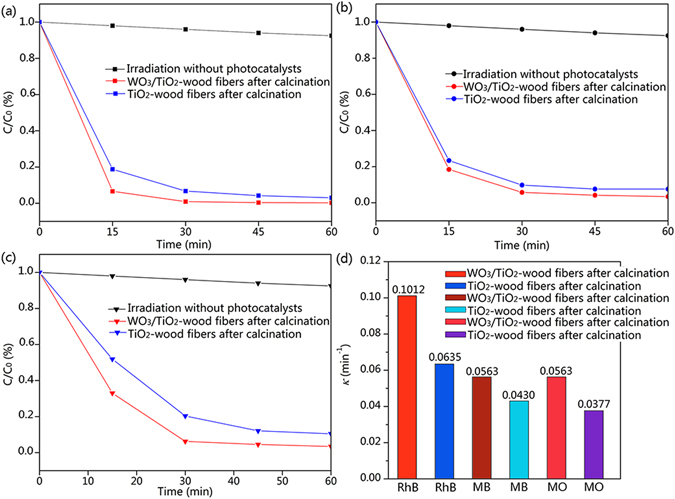
Concentration percent (*C*/*C*_*0*_) of photocatalytic (**a**) RhB, (**b**) MB and (**c**) MO with the TiO_2_-wood fibers after calcination, the WO_3_/TiO_2_-wood fibers after calcination and irradiation without photocatalysts. (**d**) First order rate constant *k* (min^−1^) of the TiO_2_-wood fibers after calcination and the WO_3_/TiO_2_-wood fibers after calcination for RhB, MB and MO.

The photocatalytic activities of the TiO_2_-wood fibers after calcination and the WO_3_/TiO_2_-wood fibers after calcination were evaluated by degradation of RhB, MB and MO under UV (wavelength <400 nm) irradiation in order to demonstrate its potential application for wastewater treatment. Figure 8(a–c) show the relationships between concentration percent (*C*/*C*_*0*_) and time for RhB, MB and MO degradation with 50 mg TiO_2_-wood fibers after calcination and 50 mg WO_3_/TiO_2_-wood fibers after calcination. And the effects of absorption of reactant by photocatalyst and photolysis of reactant were excluded by blank experiments. The WO_3_/TiO_2_-wood fibers after calcination took just 30 minutes to completely degrade RhB whereas the TiO_2_-wood fibers after calcination required 45 minutes for complete degradation of RhB. And the RhB photodegradation efficiency of the TiO_2_-wood fibers after calcination and the WO_3_/TiO_2_-wood fibers after calcination were 97.2% and 99.8%, respectively. Similarly, the WO_3_/TiO_2_-wood fibers after calcination spent only 45 minutes on complete degradation of MB and the MB photodegradation efficiency was 96.6%, whereas the TiO_2_-wood fibers after calcination spent 60 minutes and the MB photodegradation efficiency was 92.4%. Also for MO, the WO_3_/TiO_2_-wood fibers after calcination needed 45 minutes as compared to the TiO_2_-wood fibers after calcination which needed 60 minutes for complete degradation of the dye. And the MO photodegradation efficiency of the pure WO_3_/TiO_2_ after calcination and the TiO_2_-wood fibers after calcination were 96.6% and 89.6%, respectively.

Figure 8d shows the first order rate constant *k* (min^−1^) of the TiO_2_-wood fibers after calcination and the WO_3_/TiO_2_-wood fibers after calcination for RhB, MB and MO, which was calculated by the following first order equation 2 ^41^:

2 $$\mathrm{ln}({C}_{0}/C)=kt$$

where *C*_*0*_ is the initial concentration of the dye in solution and *C* is the concentration of dye at time *t*. *k* has maximum value of 0.1012 min^−1^ for RhB when the WO_3_/TiO_2_-wood fibers after calcination is used as a catalyst and it decreased to 0.0635 min^−1^ in the case of the TiO_2_-wood fibers after calcination. Furthermore, it is 0.0563 min^−1^ for the WO_3_/TiO_2_-wood fibers after calcination of MB and 0.0430 min^−1^ for the TiO_2_-wood fibers after calcination. It also shows that the value of 0.0563 min^−1^ for MO in the case of the WO_3_/TiO_2_-wood fibers after calcination as compared to the value of 0.0377 min^−1^ in the case of the TiO_2_-wood fibers after calcination. The presence of small pores on the surface of the WO_3_/TiO_2_-wood fibers after calcination (as suggested by BET), which is highly useful for a higher value of *k* (min^−1^), act as the reaction sites for the photocatalytic activity.

**Figure 9:**
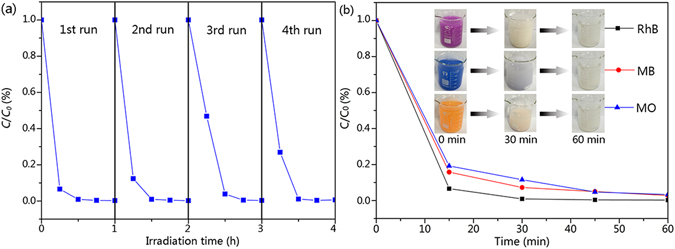
(**a**) Cycling runs in the photocatalytic degradation of RhB in the presence of 50 mg WO_3_/TiO_2_-wood fibers after calcination under UV irradiation, and (**b**) Concentration percent (*C*/*C*_*0*_) of photocatalytic RhB, MB and MO under visible light by 50 mg WO_3_/TiO_2_-wood fibers after calcination.

The WO_3_/TiO_2_-wood fibers after calcination as a kind of heterogeneous photocatalyst can be easily recycled by a simple filtration. After four recycles for the photodegradation of RhB, the catalyst did not exhibit any significant loss of activity, as shown in Fig. 9a, confirming the WO_3_/TiO_2_-wood fibers after calcination is not photocorroded during the photocatalytic oxidation of the dye pollutant. The stability of a photocatalyst is important to its practical application.

For verifying the response to the visible region, the photodegradation tests of the WO_3_/TiO_2_-wood fibers after calcination were performed by using the 50 mg catalysts for the degradation of organic dyes under visible light (500 W xenon lamp with wavelength of 420 nm). Most interestingly, the experimental results in Fig. 9b present that, for the WO_3_/TiO_2_-wood fibers after calcination, the degradation efficiency of organic dyes driven by visible light is still high, and photodegradation efficiencies for the three dyes are all over 97%, elucidating an available application of the WO_3_/TiO_2_-wood fibers after calcination under visible light. Such an important and useful property for the WO_3_/TiO_2_-wood fibers after calcination would greatly promote its application in a fast and facile elimination of organic pollutants under natural sunlight.

The result for the reactive nature of the WO_3_/TiO_2_-wood fibers after calcination towards the photocatalytic reaction is the availability of photogenerated electrons for superoxide radical generation. Also, a large surface area and pore size provide more reactive sites for reaction, which further enhance the photocatalytic efficiency of the WO_3_/TiO_2_-wood fibers after calcination. It reduces the recombination probability of photoexcited charge carriers and enhances the transport of charges.

**Figure 10:**
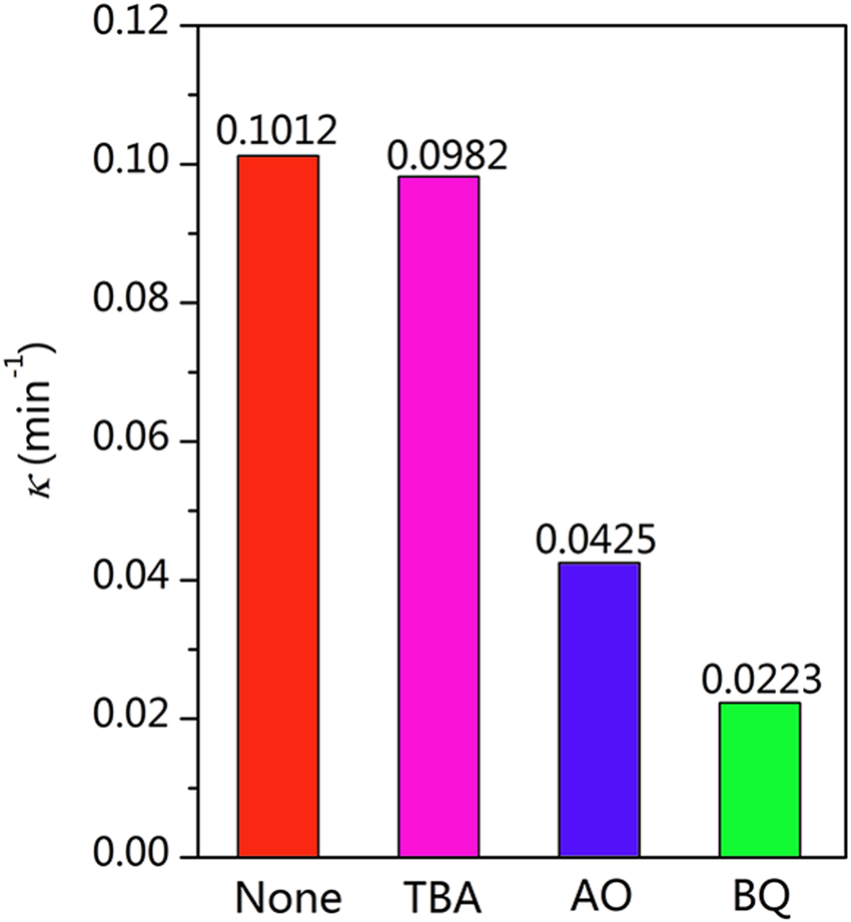
Effect of scavengers on the RhB photodegradation of WO_3_/TiO_2_-wood fibers after calcination under UV light.

The active species typically involved in the photodegradation include holes (h^+^), hydroxyl radicals (•OH) and superoxide radicals (•O_2_^−^)^42, 43^. A series of controlled experiments of RhB photodegradation under UV light were performed over the WO_3_/TiO_2_-wood fibers after calcination using different radical scavengers, as shown in Fig. 10. As a result, when TBA (scavenge •OH) is added, the degradation rate is unchanged, while the degradation rates are dramatically decreased to 22% and 42% when BQ (scavenge •O_2_^−^) and AO (scavenge h^+^) are added, respectively. These suggest that the •O_2_^−^ and h^+^ play important roles in the photodegradation process.

**Figure 11:**
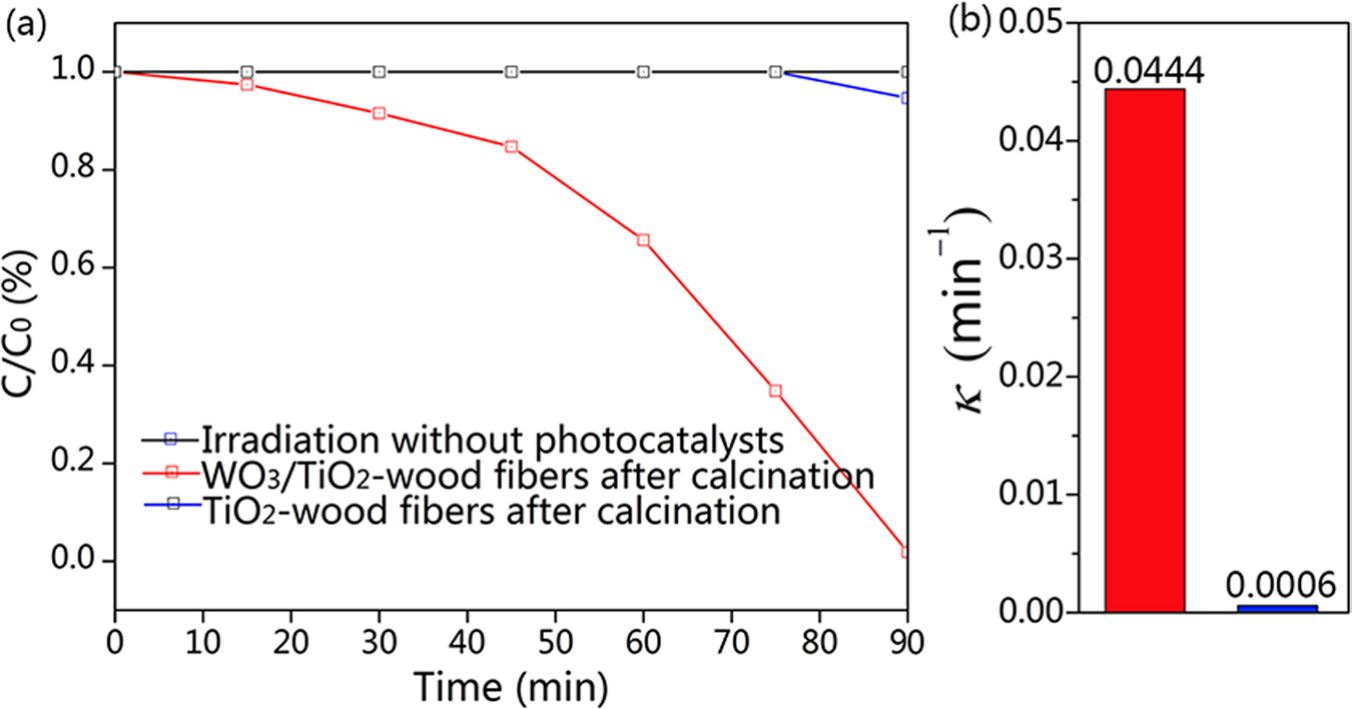
(**a**) Concentration percent (*C*/*C*_*0*_) of photocatalytic phenol with the TiO_2_-wood fibers after calcination, the WO_3_/TiO_2_-wood fibers after calcination and irradiation without photocatalysts. (**b**) First order rate constant *k* (min^−1^) of the TiO_2_-wood fibers after calcination and the WO_3_/TiO_2_-wood fibers after calcination for phenol.

To test the versatile photodegradation abilities of the samples, the photocatalytic degradation of colorless organics (phenol) has been measured. Figure 11a shows the relationships between concentration percent (*C*/*C*_*0*_) and time for phenol degradation with 50 mg TiO_2_-wood fibers after calcination, 50 mg WO_3_/TiO_2_-wood fibers after calcination, and irradiation without photocatalysts. The WO_3_/TiO_2_-wood fibers after calcination took 90 minutes to completely degrade phenol. However, the TiO_2_-wood fibers after calcination could not degrade phenol. Figure 11b shows the first order rate constant *k* (min^−1^) of the TiO_2_-wood fibers after calcination and the WO_3_/TiO_2_-wood fibers after calcination for phenol. It shows that the *k* value of 0.0444 min^−1^ for phenol in the case of the WO_3_/TiO_2_-wood fibers after calcination as compared to the value of 0.0006 min^−1^ in the case of the TiO_2_-wood fibers after calcination. The results indicate that the WO_3_/TiO_2_-wood fibers after calcination possess versatile photodegradation abilities.

**Figure 12:**
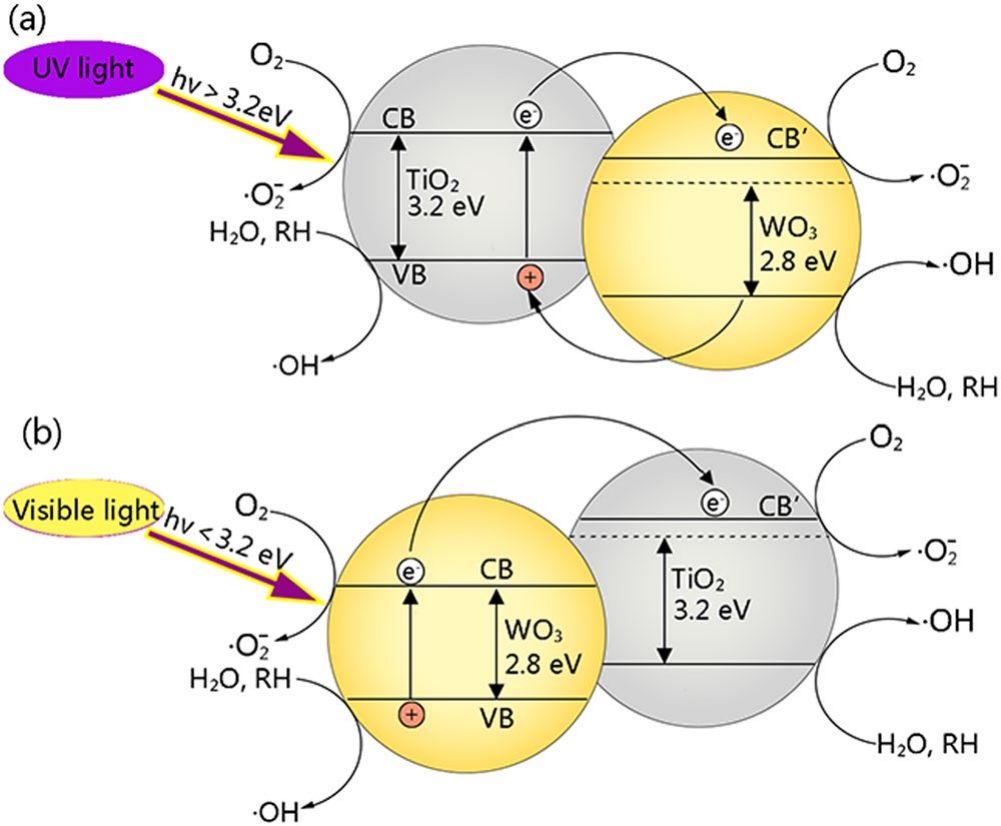
Photocatalytic mechanism schematic diagrams of the WO_3_/TiO_2_-wood fibers after calcination as (**a**) the *hν* > 3.2 eV, and (**b**) the *hν* < 3.2 eV.

Based on the above values of optical band gap energies and the photocatalytic results, we constructed the potential energy diagrams for the WO_3_/TiO_2_-wood fibers after calcination in Fig. 12. When the WO_3_/TiO_2_-wood fibers after calcination are illuminated with UV light or visible light, excited electrons are generated in the conduction band of both TiO_2_ and WO_3_. The photogenerated electrons in WO_3_ move to the conduction band of TiO_2_ easily due to the potential difference. This facile electron transfer would reduce the chance of recombination with holes formed in the valence bands of the two semiconductors. The holes migrate to the semiconductor interface either directly or after transfer from TiO_2_ to WO_3_. The reduced recombination would naturally induce photo-activity enhancement^22^.

As shown in Fig. 12a,b, the current is divided into two cases: (a) as the *hν* > 3.2 eV, that is, the photon with enough energy to excite TiO_2_. Under UV irradiation, electrons in the VB of TiO_2_ are excited into the CB of TiO_2_, and then transfer from the CB of TiO_2_ to the WO_3_, because of the lower CB of WO_3_. Furthermore, the VB edge of TiO_2_ is higher than that of WO_3_. The holes leaving in the VB of WO_3_ can move into the valence of TiO_2_, which facilitate the electron/hole separation. Thus the photo-oxidation efficiency of the sample is increased. Thus, the lower band gap of WO_3_ produces a photocatalytic effect in a wider-gap TiO_2_ by increasing the charge separation and extending the energy range of photo-excitation for the system.

(b) If a photon with not enough energy to excite TiO_2_ but is of enough energy to excite WO_3_, such as visible light, is incident (the *hν* < 3.2 eV), the hole that is created in the WO_3_ valence band is excited to the conduction band of TiO_2_, while the electron is transferred to the conduction band of TiO_2_. At this point, the electron transfer increases the charge separation and the efficiency of the photocatalytic process. After separation, the electron is free to reduce the adsorbed organic compound and the hole is available to oxidize.

In both the two cases, under light illumination, the photogenerated electron-hole pairs are produced (*hν* → e^−^ + h^+^), and holes release the captured adsorbed species by leaving behind an electron (h^+^ + O_2_^−^ → O_2_), leading a decrease of depletion layer and an increase in the conductance. At the same time, the oxygen molecules in the ambient react with the photogenerated electrons (O_2_ + e^−^ → •O_2_^−^), which creates additional photoinduced oxygen ions. The photoinduced oxygen ions are bound to the film much more weakly than the chemisorbed oxygen ions. Herein, the photoinduced oxygen ions are the crucial reactant taking parts in the following photochemical reactions^12^. When the rate of oxygen adsorption and desorption reaches to a balance, the thickness of the depletion layer and the conductivity of film gradually achieve a stable state, which can be regarded as the activated process of the depletion layer (•O_2_^−^ + *hν* → O_2_).

When the electrons and holes reach the semiconductor-environment interface, they will react with appropriate redox species (H_2_O and O_2_) to form reactive intermediates (•OH and •O_2_^−^). These radicals and photogenerated holes are extremely strong oxidants which are able to oxidize all organic materials into CO_2_ and H_2_O, leading to the degradation of organic pollutants.

**Figure 13:**
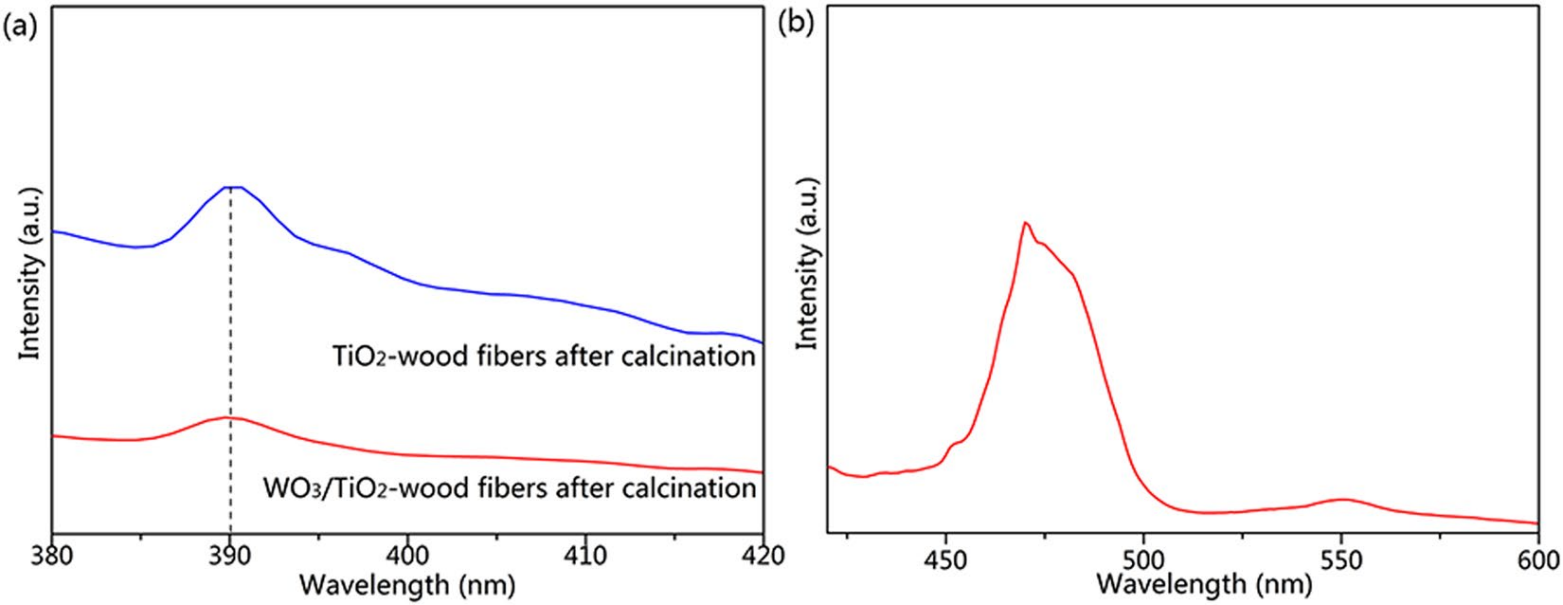
(**a**) PL spectra of the TiO_2_-wood fibers after calcination and the WO_3_/TiO_2_-wood fibers after calcination in the UV light range and (**b**) PL spectrum of the WO_3_/TiO_2_-wood fibers after calcination in the visible light range.

To test the above explanation, PL emission spectra were performed since they reveal the efficiency of charge carrier trapping, transfer, and separation of a charge carrier, and to understand the fate of electron-hole pairs in semiconductors. As WO_3_ and TiO_2_ have different emission peaks, we compared the TiO_2_-wood fibers after calcination with the WO_3_/TiO_2_-wood fibers after calcination in different wavelength ranges which are shown in Fig. 13a,b. In the UV light range (Fig. 13a), the emission peak of the TiO_2_-wood fibers after calcination appears at about 390 nm, which is in accordance with the band gap of TiO_2_ (3.2 eV). This peak is ascribed to a band–band PL emission phenomenon and excitonic PL resulting from surface oxygen vacancies and defects^44^. In the UV light range, the PL peak intensity of the WO_3_/TiO_2_-wood fibers after calcination is always lower than that of the TiO_2_-wood fibers after calcination, which indicates that the separation of photogenerated electrons and holes in the WO_3_/TiO_2_-wood fibers after calcination is more efficient than that of the TiO_2_-wood fibers after calcination. In another word, the photogenerated charge recombination of the WO_3_/TiO_2_-wood fibers after calcination is inhibited^45^. Moreover, in Fig. 13b, the emission at 470 nm corresponding to 2.5 eV, which is approximately equal to the band-gap of the WO_3_/TiO_2_-wood fibers after calcination, can be due to the transition from the conduction band of WO_3_ to the conduction band of TiO_2_.

## Conclusions

Herein, we combined a two-steps hydrothermal method and a calcination process to fabricate a heterostructured WO_3_/TiO_2_ photocatalysts from wood fibers. The wood fibers acted as carbon substrates to prepare the WO_3_/TiO_2_ photocatalysts with high surface area and unique morphology. The prepared WO_3_/TiO_2_-wood fibers after calcination exhibit higher potential for application as an UV light or a visible light photocatalyst for degradation of organic pollutants. These studies indicate that the WO_3_/TiO_2_-wood fibers after calcination with high surface area and large aspect ratio can provide more reactive sites for photocatalytic. It reduces the recombination probability of photoexcited charge carriers and also increases the transport of charges. For purpose of utilizing of solar energy, we anticipate that the WO_3_/TiO_2_-wood fibers photocatalysts could be a promising photocatalyst to decompose the harmful chemicals existed in the environment.

## Methods

### Materials

All chemicals supplied by Shanghai Boyle Chemical Company, Limited were of analytical reagent-grade quality and used without further purification. Deionized water was used throughout the study. Wood fibers were obtained from poplar wood (*Populus ussuriensis* Kom), which is one of the most common tree species in the northeast of China. The wood fibers were oven-dried (24 h, 103 ± 2 °C) to constant weight after ultrasonically rinsing in deionized water for 30 min.

Ammonium fluorotitanate (0.4 M) and boric acid (1.2 M) were dissolved in distilled water at room temperature under vigorous magnetic stirring. Then, a solution of 0.3 M hydrochloric acid was added until the pH reached approximately 3. 75 mL of the adjusted solution and 5 g wood fibers were transferred into a 100 mL Teflon container. The autoclave was sealed and maintained at 90 °C for 5 h, then allowed to naturally cool to room temperature. Finally, the TiO_2_-wood fibers were washed with distilled water and absolute ethanol for several times, dried in an oven. Thus, the TiO_2_-wood fibers were obtained.

The synthetic route of the WO_3_/TiO_2_-wood fibers was as follows. Firstly, the 1.8 g of Na_2_WO_4_·2H_2_O was dissolved into 100 ml mixed precursor solution (containing 20 ml absolute ethanol and 80 ml distilled water) at room temperature, followed by being acidified to 1.0 of pH value using the H_2_SO_4_ solution. The mixed solution was transferred into a stainless steel autoclave. Then the TiO_2_-wood fibers were soaked into the reaction solution. The autoclave was sealed and maintained at 110 °C for 24 h, and then cooled down to room temperature. Finally, the composite films were formed on the wood fibers and then washed with distilled water and absolute ethanol for several times, and dried in an oven. Thus the WO_3_/TiO_2_-wood fibers were obtained and the C content in the production was 36.6%. In both cases, the samples were calcined in flowing air at 500 °C for 3 h. For comparison, the pure WO_3_/TiO_2_ without wood fibers after calcination was prepared.

### Characterization

The morphology and microstructure were characterized by field-emission scanning electron microscopy (FE-SEM, JSM-7500F, JEOL, Japan) operating at 12.5 kV. The crystal structure of the as-prepared product was investigated by X-ray diffraction (XRD, Bruker D8 Advance, Germany) with Cu Kα radiation of wavelength λ = 1.5418 Å, using a step scan mode with the step size of 0.02° and a scan rate of 4° min^−1^, at 40 kV and 40 mA ranging from 5° to 80°. Further evidence for the composition of the product was inferred from the X-ray photoelectron spectroscopy (XPS, Thermo ESCALAB 250XI, USA), using an ESCALab MKII X-ray photoelectron spectrometer with Mg-Kα X-rays as the excitation source. Thermogravimetric and Differential Thermal Analysis (TG–DTA) spectra were performed using a PE-TGA7 thermogravimetric analyzer (Perkin Elmer Company) and a DTA/9050311 high temperature differential analyzer. 10 mg of the samples were taken and measured in air, and then treated in 150 ml/min of dry pure N_2_ with temperatures at the rate of 10 C/min ranging from 20 °C to 800 °C. Specific surface areas of the prepared products were measured by the Brunauer–Emmett–Teller (BET) method based on N_2_ adsorption at the liquid nitrogen temperature using a 3H-2000PS2 unit (Beishide Instrument S&T Co., Ltd). Optical properties of the materials were characterized by the UV-vis diffuse reflectance spectroscopy (UV-vis DRS, Beijing Purkinje TU-190, China) equipped with an integrating sphere attachment, which BaSO_4_ was the reference. Photoluminescence (PL) emission spectra were used to investigate the fate of photogenerated electrons and holes in the sample, and were recorded on a FluoroMax 4 fluorescence spectrometer (HORIBA Jobin Yvon Company, France). The excitation wavelength was 350 nm with the scanning speed of 600 nm·min^−1^. The widths of both excitation slit and emission slit were 10 nm.

### Photocatalytic test

For photocatalytic tests, a certain amount of sample was dissolved in 100 ml aqueous solutions of rhodamine B (RhB), methylene blue (MB), methyl orange (MO) or phenol in glass beakers. The concentration of RhB, MB and MO was 10 mg in 1 L of H_2_O, while concentration of the aqueous phenol solution was 67.2 mg/L. At first, the solution was stirred continuously in the dark for 60 minutes to establish adsorption–desorption equilibrium among the photocatalysts and dye solution, then this solution was brought into UV irradiation. A 500 W ultraviolet lamp with the wavelength range of 425 nm was used as light source. Then, the glass beaker was placed in front of the lamp during continuous magnetic stirring. 5 ml of solution was collected and centrifuged. Then UV absorption measurements were used to observe the photodegradation at specific time intervals. The absorption peaks for RhB, MB, MO and phenol were observed at 553, 664, 464 and 270 nm respectively. For stability measurements the same materials were taken from the solution and the above mentioned steps were repeated for three times. Moreover, to evaluate the role of different active species in the photocatalytic reaction, controlled experiments using different radical scavengers including ammonium oxalate (AO), tert-butyl alcohol (TBA) and 1,4-benzoquinone (BQ) were performed under UV light to scavenge the h^+^, •OH and •O_2_^−^ species, respectively^42^.
